# A closed model for the respiratory system in mammals

**DOI:** 10.1186/1471-2202-13-S1-P51

**Published:** 2012-07-16

**Authors:** Choongseok Park, Yaroslav Molkov, Alona Ben-Tal, Natalia Shevtsova, Jeffrey Smith, Ilya Rybak, Jonathan Rubin

**Affiliations:** 1 Department of Mathematics, University of Pittsburgh, Pittsburgh, PA15260, USA; 2 Indiana University - Purdue University Indianapolis, Department of Mathematical Sciences, Indianapolis, IN 46202, USA; 3 Institute of Information and Mathematical Sciences, Massey University, Auckland, New Zealand; 4 Drexel University College of Medicine, Philadelphia, PA 19129, USA; 5 Cellular and Systems Neurobiology Section, Laboratory of Neural Control, NINDS, Bethesda, MD 20892-3700, USA

## 

The interactions between rhythmogenic neuronal networks and physiological systems are crucial components in understanding how a system maintains homeostasis under diverse circumstances. Various aspects of such interactions should be addressed including how rhythms are generated in neuronal networks, how feedback signals interact with neuronal networks to regulate levels of important physiological quantities, and how changes in demands on the system can be handled in a way that restores homeostasis.

In this work, we explore these issues in the context of neuronal networks in the mammalian brainstem that generate the respiratory rhythm when isolated from the physiological systems that drive actual breathing [1]. These networks include neuronal components within the Bötzinger (BötC) and pre-Bötzinger (pre-BötC) complexes and the retrotrapezoid nucleus/parafacial respiratory group (RTN/pFRG). Experimental results on respiratory rhythm generation have suggested that neurons within the BötC and pre-BötC are responsible for the generation of primary respiratory rhythms, which are modulated by oscillations in the RTN/pFRG. Previous computational work has elucidated a possible set of mechanisms through which these brain stem neurons interact and produce a variety of experimental results [2].

Separate past work has developed a physiologically detailed yet tractable model of respiratory physiology [3]. This model is comprised of a very simplified version of a neuronal network oscillator that generates respiratory rhythms, an integrator that delivers respiratory output , and lung components responsible for air exchange. This earlier study considered reasonable ways to link the neuronal oscillator/integrator with these physiological components in a closed loop feedback system to achieve respiratory frequency and amplitude control.

In our work, we simulate and analyze a model that links the detailed neuronal rhythm generator with the physiological lung components. We consider the roles of mechanical and chemoreceptive feedback signals in maintaining stable respiratory rhythms and in responding to metabolic or environmental perturbations. In particular, we study the response to hypercapnia, or excessive carbon dioxide [4]. The idea is that excessive exposure to CO2 may lead to CO2 accumulation in the blood, which will recruit certain neuronal elements in the RTN/pFRG and alter the drive to core rhythmogenic elements. Changes in rhythmic output patterns activate certain muscles that alter breathing in a way that restores CO2 homeostasis. We analyze the roles of neurons’ intrinsic dynamic features, the architecture and strengths of synaptic connections among neurons, and the roles of particular feedback systems in achieving baseline rhythms and appropriate responses to perturbations.
